# *Ddx1* knockout results in transgenerational wild-type lethality in mice

**DOI:** 10.1038/srep09829

**Published:** 2015-04-24

**Authors:** Matthew R. Hildebrandt, Devon R. Germain, Elizabeth A. Monckton, Miranda Brun, Roseline Godbout

**Affiliations:** 1 Department of Oncology, University of Alberta, Cross Cancer Institute, 11560 University Avenue, Edmonton, Alberta T6G 1Z2, Canada

## Abstract

DEAD box 1 (DDX1) is a member of the DEAD box family of RNA helicases which are involved in all aspects of RNA metabolism. DDX1 has been implicated in a variety of biological processes, including 3’-end processing of mRNA, DNA repair, microRNA processing, tRNA maturation and mRNA transport. To study the role of DDX1 during development, we have generated mice carrying a constitutive *Ddx1* knock-out allele. *Ddx1*^+/−^ mice have no obvious phenotype and express similar levels of DDX1 as wild-type mice indicating compensation from the intact *Ddx1* allele. Heterozygote matings produce no viable *Ddx1^−/−^* progeny, with *Ddx1*^−/−^ embryos dying prior to embryonic day (E) 3.5. Intriguingly, the number of wild-type progeny is significantly decreased in heterozygote crosses, with two different heterozygote populations identified based on parental genotype: (i) normal *Ddx1*^+/−^ mice which generate the expected number of wild-type progeny and (ii) Ddx1*^/−^ mice (with * signifying a non-genetically altered allele) which generate a significantly reduced number of wild-type mice. The transgenerational inheritance of wild-type lethality observed upon crossing Ddx1*^/−^ mice is independent of parental sex and occurs in *cis* through a mechanism that is different from other types of previously reported transgenerational epigenetic inheritance.

DEAD box proteins are RNA unwinding proteins that are characterized by 12 conserved motifs, including the signature motif, D(asp)-E(glu)-A(ala)-D(asp) which is involved in ATP hydrolysis. These proteins have been implicated in all aspects of RNA metabolism including transcription, transport, translation, and degradation^1^,^2^,^3^,^4^. Most DEAD box proteins unwind RNA-RNA duplexes *in vitro* through localized strand destabilization rather than processive unwinding.^5^,^6^ DEAD box proteins have been shown to be modulators of ribonucleoprotein complexes by displacing or recruiting different proteins to these complexes^6^,^7^.

DEAD box 1 (DDX1) was first identified by differential screening of a retinoblastoma cDNA library, and subsequently found to be amplified and overexpressed in a subset of retinoblastoma and neuroblastoma tumours and cell lines^8^,^9^,^10^,^11^,^12^. DDX1 expression is ubiquitous, with proliferating cells and cells derived from neuroectodermal tissues expressing the highest levels of DDX1^8^,^13^. DDX1 is predominantly located in the nucleus of non-*DDX1-*amplified normal and cancer cells^14^. However, when amplified and overexpressed, elevated levels of DDX1 are observed in both the nucleus and cytoplasm^15^. In breast cancer, DDX1 is a negative prognostic indicator when overexpressed or mis-localized to the cytoplasm^16^,^17^.

DDX1 has been associated with a number of biological processes both in the nucleus and the cytoplasm. In the nucleus, DDX1 forms foci that co-localize with cleavage bodies and reside adjacent to Cajal bodies and gems, three spatially related RNA processing bodies.^14^,^18^ When cells are exposed to ionizing radiation, DDX1 is recruited to sites of DNA double-strand breaks where it co-localizes with DNA damage response proteins^19^. DDX1 is also part of the tRNA ligase complex involved in pre-tRNA processing, and the pri-miRNA microprocessor complex involved in the processing of miRNAs^20^,^21^,^22^,^23^. In the cytoplasm, DDX1 is found in RNA containing granules involved in the transport of RNAs in neurons, as well as stress granules^24^,^25^,^26^.

Although it is possible to knockdown DDX1 in immortalized cancer cell lines and normal fibroblast cultures^19^, to date there have been no reports of DDX1 null cell lines. Furthermore, our attempts to knockout *DDX1* in HeLa cells using CRISPR/Cas9 technology have been unsuccessful. In fact, whereas 2-3 rounds of CRISPR/Cas9 transfection resulted in a 50% reduction in DDX1 levels, repeated rounds of transfection (up to 10) generated cells with near normal levels of DDX1. Based on these data, it appears that HeLa cells have a compensatory mechanism in place to prevent long-term reduction in DDX1 levels.

We have generated *Ddx1* heterozygous mice that contain a constitutive gene-trapped allele. Here, we show that *Ddx1*^−/−^ embryos die during the pre-blastocyst stage of development. Intriguingly, the ratio of wild-type to heterozygote mice is significantly reduced in heterozygote intercrosses, with wild-type progeny dying between E3.5 and E6.5. By tracing parental lineages, we identified a subpopulation of heterozygous mice that generate significantly reduced numbers of wild-type progeny. This phenotype is observed in both FVB and C57BL/6 backgrounds, and is transmitted through both sexes. Analysis of the methylation status of the *Ddx1* gene revealed no differences between the heterozygous and wild-type mice.

## Results

### Ddx1^−/−^ embryos die pre-implantation

**Figure 1:**
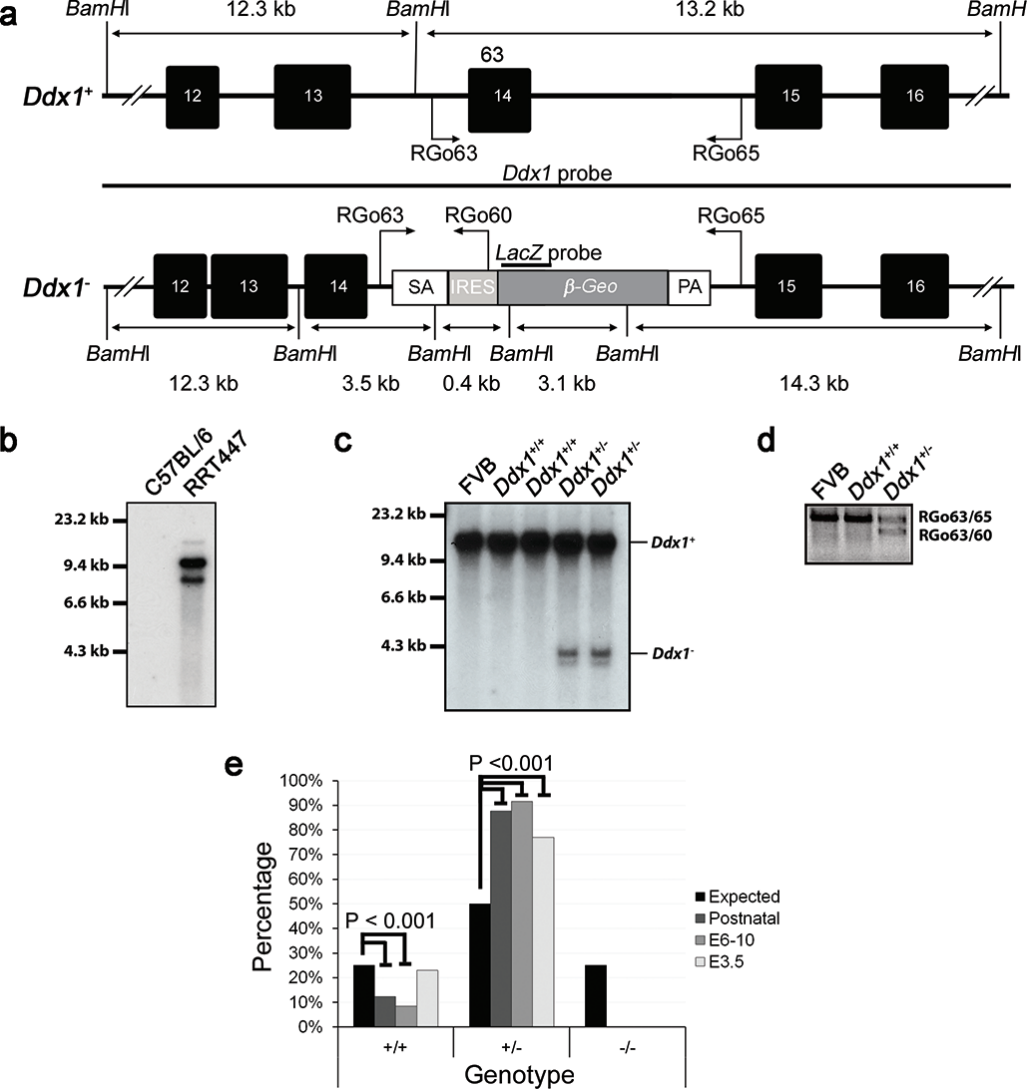
Genomic map of the gene-trap insertion site. ESCs containing a single gene-trap insertion in *Ddx1* were purchased from BayGenomics. (a) The insertion containing a *β-geo* gene, splice acceptor (SA) and a polyadenylation signal (PA) is located between exons 14 and 15 of *Ddx1*. The insertion generates a truncated DDX1 protein fused to LacZ. Locations of primers (RGo) and Southern blot probes used for genotyping are also shown. (b) Southern blot analysis of RRT447 cell line using a ^32^P-labeled cDNA probe specific to *β*-geo. (c) Southern blot analysis of wild-type and *Ddx1*^+/−^ mice using a ^32^P-labeled cDNA probe specific to *Ddx1*. (d) PCR amplification of genomic DNA for routine genotyping using primers shown in (A). (e) Progeny from heterozygous intercrosses (*Ddx1*^+/−^ or *Ddx1*^+/−^) were collected and genotyped at different developmental stages. No *Ddx1*^−/−^ progeny were observed out of a total of 758 postnatal offspring, 225 E6-10 embryos and 91 E9.5 blastocysts genotyped. A significant decrease in the percentage of wild-type mice was observed post E3.5 (P < 0.001). Fisher’s exact tests were performed to determine significant differences between the expected and observed ratios of *Ddx1*^+/+^ to *Ddx1*^+/−^ mice.

A mouse embryonic stem cell (ESC) line containing an intronic gene trap in the *Ddx1* gene [*GT*(RRT447)Byg; abbreviated as RRT447] was obtained from BayGenomics. Insertion of the gene-trap was in intron 14 of *Ddx1*. RRT447 ES cells were microinjected into blastocysts to generate male chimeras which were mated to C57BL/6 females to obtain germ-line transmission of the *Ddx1^GT^*^(RRT447)Byg ^allele, designated *Ddx1*^-^ (Figure 1a). Southern blot analysis with cDNA probes to *β-geo* and *Ddx1* exons 10-17 showed the presence of a single gene-trap in the RRT447 ESCs and in the *Ddx1^+/−^*mice generated from the ESCs (Figures 1b-c). *Ddx1*^+/−^ mice showed no phenotypic abnormalities and produced phenotypically normal pups.

Offspring produced by heterozygote intercrosses were genotyped by PCR to identify both wild-type and gene-trap *Ddx1* alleles. Of 408 weaned pups analyzed, no *Ddx1*^−/−^ pups were identified (Figure 1e). Next, we genotyped embryos from both pre-implantation (E3.5) and post-implantation (E10) stages. Again, no *Ddx1*^−/−^ embryos were identified at either stage, indicating that *Ddx1*^−/−^ embryos die pre-implantation.

### Two distinct populations of Ddx1^+/−^ mice produce differing ratios of wild-type to heterozygous progeny

The expected ratio of wild-type to heterozygote animals in *Ddx1* heterozygous intercrosses is 1 wild-type to 2 heterozygotes, as no *Ddx1* knockouts survive to E3.5. Intriguingly, analysis of all heterozygote matings revealed a considerable deviation from the expected 1:2 ratio, with an observed ratio of 1:9 (Table 1). To rule out the possibility of a recessive lethal mutation linked to the wild-type *Ddx1* allele in the C57BL/6 background, *Ddx1*^+/−^ mice were backcrossed for six generations to wild-type FVB mice. When FVB *Ddx1*^+/−^ mice were intercrossed, we obtained a similar genotype ratio as in the C57BL/6 background (Table 1). In total, 292 weaned pups in the FVB background were genotyped by PCR analysis, with an observed ratio of 1 wild-type to 7 heterozygous mice. As both the FVB and C57BL/6 strains generated the same wild-type lethality phenotype, subsequent analyses were carried out using both the FVB and C57BL/6 *Ddx1* lines.

**Figure 2:**
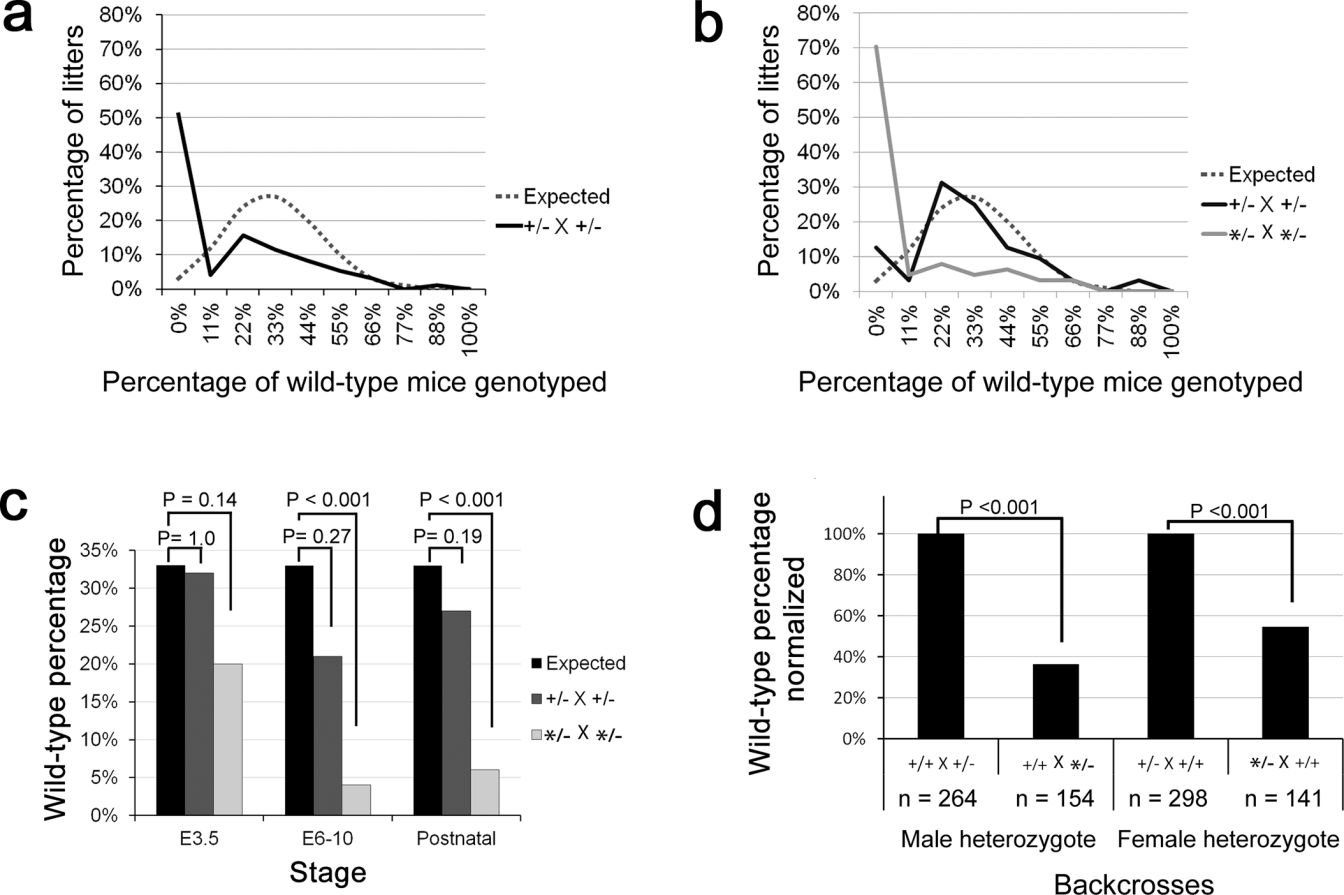
Heterozygous mice generate a bimodal distribution of progeny genotypes. (a) Litters from heterozygous intercrosses (*Ddx1*^+/−^ or *Ddx1*^+/−^) that contained at least 5 pups were plotted as a percentage of wild-type mice generated (n = 178). A normal random distribution plotted around the expected value of 33% wild-type is included for comparison. (b) *Ddx1*^+/−^ and Ddx1*^/−^ intercrosses were separated (n = 32 and n = 146, respectively) and the percentage of wild-type mice generated was plotted. (c) The percentage of wild-type mice at ages E3.5, E6-10 and P0 from *Ddx1*^+/−^ (n = 22, 61, 229, respectively) or Ddx1*^/−^ (n = 69, 164, 529, respectively) intercrosses were plotted against the expected percentage. (d) Backcrosses (wild-type X heterozygote) from both FVB and C57BL/6 (combined) mice were separated by genotype and sex of the heterozygote. The percentage of wild-type genotyped was normalized to the *Ddx1*^+/−^ backcross. Fisher’s exact tests were performed to determine significant differences.

Analysis of *Ddx1*^+/+^ *to Ddx1*^+/−^ progeny ratios in individual litters of *Ddx1* heterozygous intercrosses revealed a bimodal distribution, suggesting the possibility of two distinct heterozygote populations (Figure 2a). Upon more detailed examination of individual litters, we discovered that a normal ratio of wild-type to heterozygous progeny was consistently observed when *Ddx1*^+/−^ animals were derived from *Ddx1*^+/+^ X *Ddx1*^+/−^ backcrosses (Figure 2b). In contrast, *Ddx1*^+/−^ animals derived from *Ddx1*^+/−^ X *Ddx1*^+/−^ intercrosses generated significantly fewer wild-type progeny. To distinguish the two *Ddx1*^+/−^ populations, we designated the *Ddx1*^+ ^allele inherited from *Ddx1*^+/− ^X *Ddx1*^+/−^ intercrosses as Ddx1* and heterozygous mice derived from these crosses as Ddx1*^/−^.

### Ddx1*-associated lethality occurs between E3.5 and E6.5

To further characterize Ddx1*-associated lethality, we carried out heterozygote intercrosses using: (i) heterozygote male and female mice generated from *Ddx1^+/+^* X *Ddx1^+/−^* matings (Ddx1^+/−^), and (ii) heterozygote male and female mice generated from *Ddx1^+/−^* X *Ddx1^+/−^* matings (Ddx1*^/−^). Genotyping the progeny of heterozygote intercrosses at different stages of development revealed reduced numbers of Ddx1*^/^* progeny at E6.5 and later (Figure 2c). Only ~5% of the progeny generated at E6.5 in Ddx1*^/−^ intercrosses were wild-type (Ddx1*^/^*). As no further reduction in wild-type (Ddx1*^/^*) progeny numbers were observed after E6.5, we conclude that the lethality observed in Ddx1*^/^* embryos is occurring pre-E6.5.

To further define when Ddx1*^/^* mice die, E3.5 blastocysts were genotyped. Ratios of both *Ddx1*^+/+^ to *Ddx1*^+/−^ and Ddx1*^/^* to Ddx1*^/−^ were normal at E3.5, suggesting that Ddx1*^/^* lethality occurs during the post-blastocyst stages of development. The most likely causes for the observed lethality are therefore failure to implant or failure to continue development post-implantation. As no reabsorbed embryos were observed in Ddx1*^/−^ intercrosses, lethality is likely due to a failure to implant.

In the heterozygote intercrosses described above, wild-type lethality was observed in Ddx1*^/^* progeny*.* In order to address whether a single Ddx1* allele can give rise to lethality, both *Ddx1*^+/−^ and Ddx1*^/−^ mice were backcrossed to *Ddx1^+/+^* mice*.* As expected, *Ddx1*^+/−^ backcrosses (producing *Ddx1*^+/+ ^and *Ddx1*^+/−^ offspring) yielded the expected number of wild-type progeny. In contrast, Ddx1*^/−^ backcrosses (producing Ddx1*^/+ ^and *Ddx1*^+/−^ offspring) yielded approximately 40% of the expected number of wild-type mice, indicating reduced viability in Ddx1*^/+ ^animals.

### Inheritance of the Ddx1* allele is parental sex independent

As *Ddx1*^+/− ^mice can be generated from Ddx1*^/− ^x *Ddx1^+/+^*crosses, we can infer that the modification responsible for the observed lethality must be linked with the specific *Ddx1* allele, rather than transmitted in *trans*. In addition, since inheritance of the modified Ddx1* allele occurs in both heterozygous (Ddx1*^/−^) and homozygous wild-type (Ddx1*^/^*) progeny, the allele must be transgenerationally maintained. The most common form of epigenetic transgenerational modification in mice is genomic imprinting. Genomic imprinting involves methylation based silencing which occurs during gamete formation, and is sex asymmetric.

While *Ddx1* has not previously been reported to undergo genomic imprinting, the observed lethality could be explained by an imprinting mechanism of inheritance. In order to determine if genomic imprinting is responsible for modulating *Ddx1*, progeny generated from heterozygote female or male backcrosses were analyzed. Analysis of 295 progeny from Ddx1*^/−^ backcrosses (154 offspring from male Ddx1*^/−^ mice and 141 from female Ddx1*^/−^ mice) revealed altered wild-type to heterozygote ratios in progeny generated from both male and female Ddx1*^/−^ mice (Figure 2d). The lack of a sex-specific effect indicates that traditional genomic imprinting is not responsible for the observed lethality.

### Expression compensation at the Ddx1 loci

**Figure 3:**
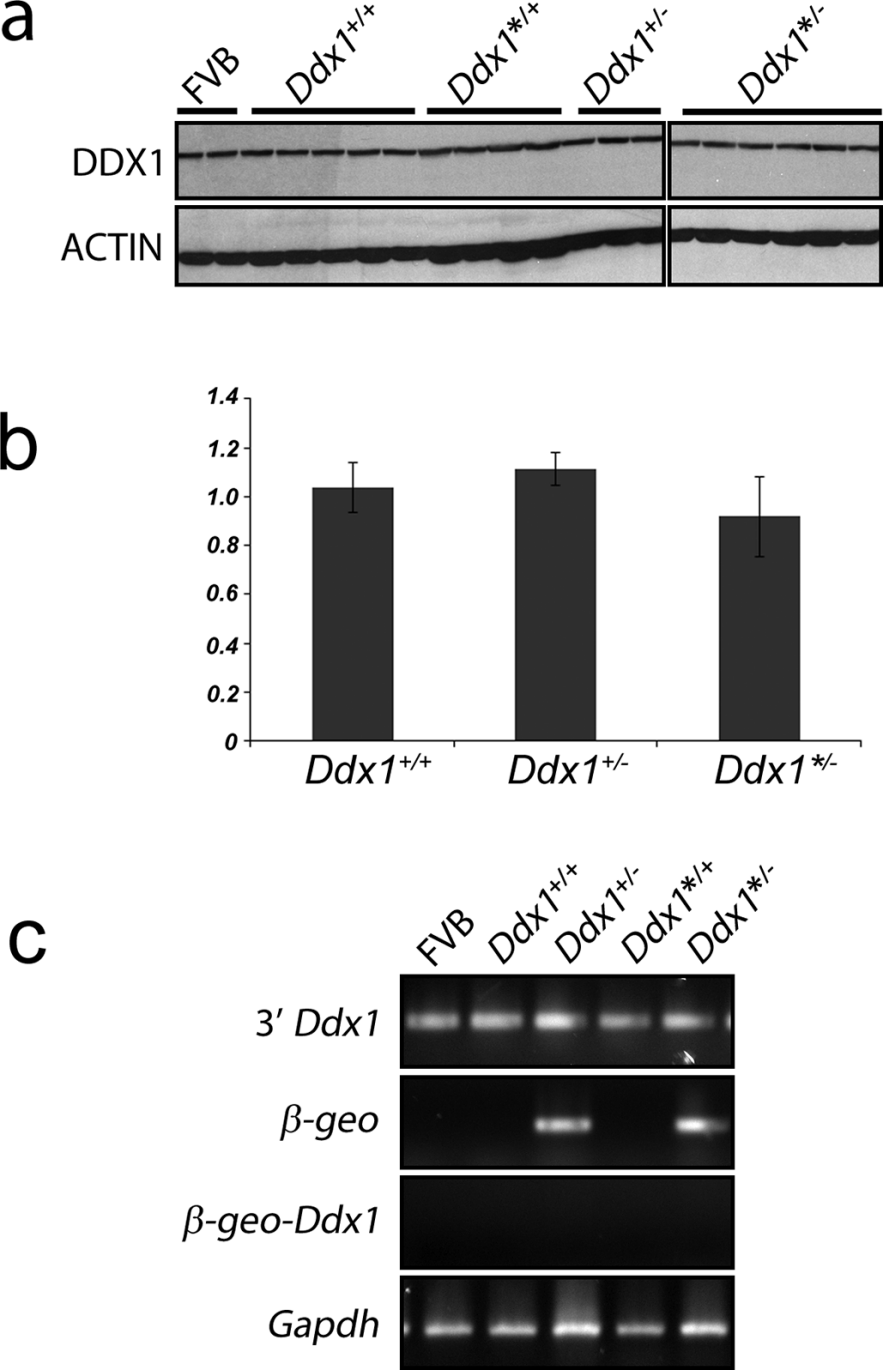
*Ddx1* mRNA and protein expression levels are similar in wild-type and heterozygous animals. (a) Western blot analysis of 50 μg of whole brain lysates from P0-3 mice of the indicated genotypes. Blots were immunostained with anti-DDX1 antibody (top) and anti-actin antibody (bottom). (b) Quantitative real-time PCR of P0-3 mouse brain RNA from the indicated genotypes. qPCR was carried out with 3’ *Ddx1* primers and *Gapdh* primers as a control (n≥4 for each sample). Expression levels for *Ddx1* are plotted relative to wild-type. Error bars show standard error of the mean. (c) Semi-quantitative RT-PCR analysis of cDNAs generated from P0-3 mouse brain RNA. cDNA samples were amplified with *Ddx1* primers 3’ to the gene-trap (top panel), primers specific to *β-geo* (second panel), primers to the 3’ end of *β-geo* and the 3’ region of *Ddx1* (third panel), and primers to *Gapdh* as a control (bottom panel).

Western blot analysis of brain tissues using anti-DDX1 antibody showed similar levels of DDX1 in all progeny irrespective of genotype (Figure 3a). The absence of truncated DDX1 products in heterozygote mouse brain using an antibody prepared against the N-terminus of DDX1 suggests that stable DDX1 protein is not produced from the *Ddx1* gene-trap allele. In agreement with western blot data, qPCR analysis of *Ddx1^+/+^*, *Ddx1^+^*^/−^ and Ddx1*^/− ^mice showed similar *Ddx1* mRNA levels (Figure 3b).

To further address expression from the wild-type *Ddx1* and gene-trap alleles, we carried out RT-PCR using mouse brain RNA isolated from each genotypic group (*Ddx1^+/+^*, *Ddx1^+^*^/−^, Ddx1*^/^*, Ddx1*^/−^, and FVB control). PCR amplification of *Ddx1* transcripts (exons 22-26) generated a positive signal for *Ddx1* in all samples, and β*-geo* transcripts were detected in all samples containing the gene-trapped *Ddx1* allele*.* These results indicate that *Ddx1* is biallelically expressed in heterozygous mice (Figure 3c). RT-PCR analysis using a 5’ primer specific to the gene trap and a 3’ primer specific to *Ddx1* (exon 21)*,* downstream of the gene trap region, failed to produce a signal, indicating that the gene trap transcript is not being spliced into the downstream region of *Ddx1* (Figure 3c). These results indicate compensation from the one functional *Ddx1* allele in heterozygous mice, resulting in similar levels of DDX1 in heterozygous and wild-type mouse brain. Similar results were obtained in liver (data not shown).

### DNA methylation is not altered in the Ddx1* allele

*Ddx1* compensation in heterozygous mice likely arises from changes in gene transcription as *Ddx1* RNA levels are similar in wild-type and heterozygous mice. While we previously showed that genomic imprinting is not likely to be responsible for the phenotypes observed, it remains possible that DNA methylation is the mechanism by which the Ddx1* allele is modified. CpG methylation of promoter regions is commonly associated with alterations in gene expression. Low levels of transcription are generally associated with increased methylation. Importantly, altered methylation patterns can potentially be inherited, leading to the observed transgenerational nature of genomic imprinting.

**Figure 4:**
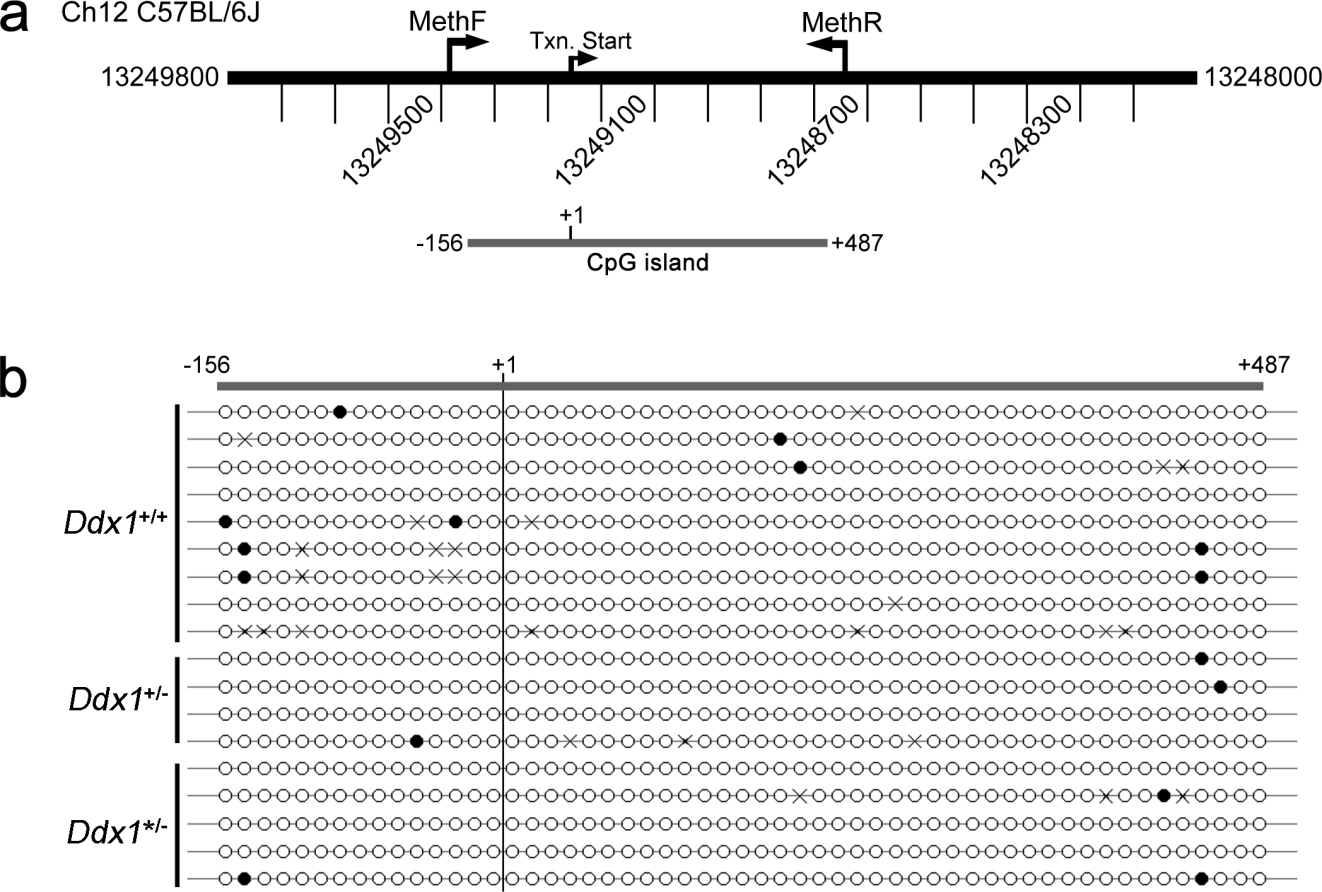
Methylation analysis at the *Ddx1* transcription start site. (a) A CpG island consisting of 55 CpG sites was predicted flanking the transcription (txn) start site of *Ddx1* from -156 to +487*.* MethF and MethR indicate binding sites of primers used to amplify the region following bisulfite conversion. (b) A lollipop diagram shows the methylation status of each of the 55 CpGs, where a white circle indicates no methylation and a black circle indicates methylation. At least 4 clones from each genotype (*Ddx1*^+/+^, *Ddx1*^+/−^, and Ddx1*^/−^) were analyzed for their methylation patterns. A cross indicates indeterminate methylation.

Using MethPrimer prediction software we identified a single CpG island in the *Ddx1* gene^27^. This CpG island contains 55 CpGs and flanks the *Ddx1* transcriptional start site from –156 to +487 bp (Figure 4a). Using bisulfite conversion of genomic DNA followed by DNA sequencing, we analyzed DNA methylation patterns in *Ddx1*^+/+^, *Ddx1*^+/−^ and Ddx1*^/− ^mice. At least 4 clones from each group were sequenced. No differences in methylation patterns were observed between the three different groups indicating that DNA methylation is likely not the mechanism regulating *Ddx1* gene compensation or Ddx1* allele modification (Figure 4b).

## Discussion

Germ-line knockout of a number of DEAD box genes, including *Ddx5*, *Ddx11*, *Ddx20* and *Ddx58,* results in embryonic lethality in mice^28^,^29^,^30^,^31^,^32^. Other DEAD box gene knockout mice are viable but have defects in gametogenesis; e.g., germ-line knockout of *Ddx4* (*Vasa*) and *Ddx25* both result in spermatid maturation defects^33^,^34^. The earliest stage lethality upon knockout of a DEAD box gene was observed in *Ddx20* (*DP103, Gemin)* knockout mice. *Ddx20*^−/−^ mice die at the 2-cell stage when zygotic gene expression is activated after rapid degradation of maternal RNAs (referred to as maternal to zygote transition or MZT). DDX20 is up-regulated in the 2-cell stage embryo and has been postulated to be involved in the reprogramming that occurs during maternal to zygote transition^31^,^35^.

*Ddx1*^−/−^ mice die pre-E3.5 suggesting an essential role for DDX1 in early embryonic development. In light of DDX1’s demonstrated roles in RNA binding, RNA/RNA unwinding and RNA transport^19^,^24^,^26^, loss of DDX1 may affect the secondary structure, stability, degradation, subcellular localization and/or translation of RNAs. It is therefore possible that DDX1 plays a similar role to that proposed for DDX20 in the reprogramming from maternal RNA utilization to active transcription from the zygote genome. Lethality could result from disruption of maternal RNA degradation which would interfere with zygote genome activation. Alternatively, deregulation of newly-synthesized zygotic transcripts could have lethal consequence for the developing embryo. The early embryonic lethality associated with *Ddx1* and *Ddx20* knock-out suggests distinct roles for these two genes, as expression of DDX1 at early embryonic stages does not compensate for *Ddx20*^−/−^ lethality and vice versa.

**Figure 5:**
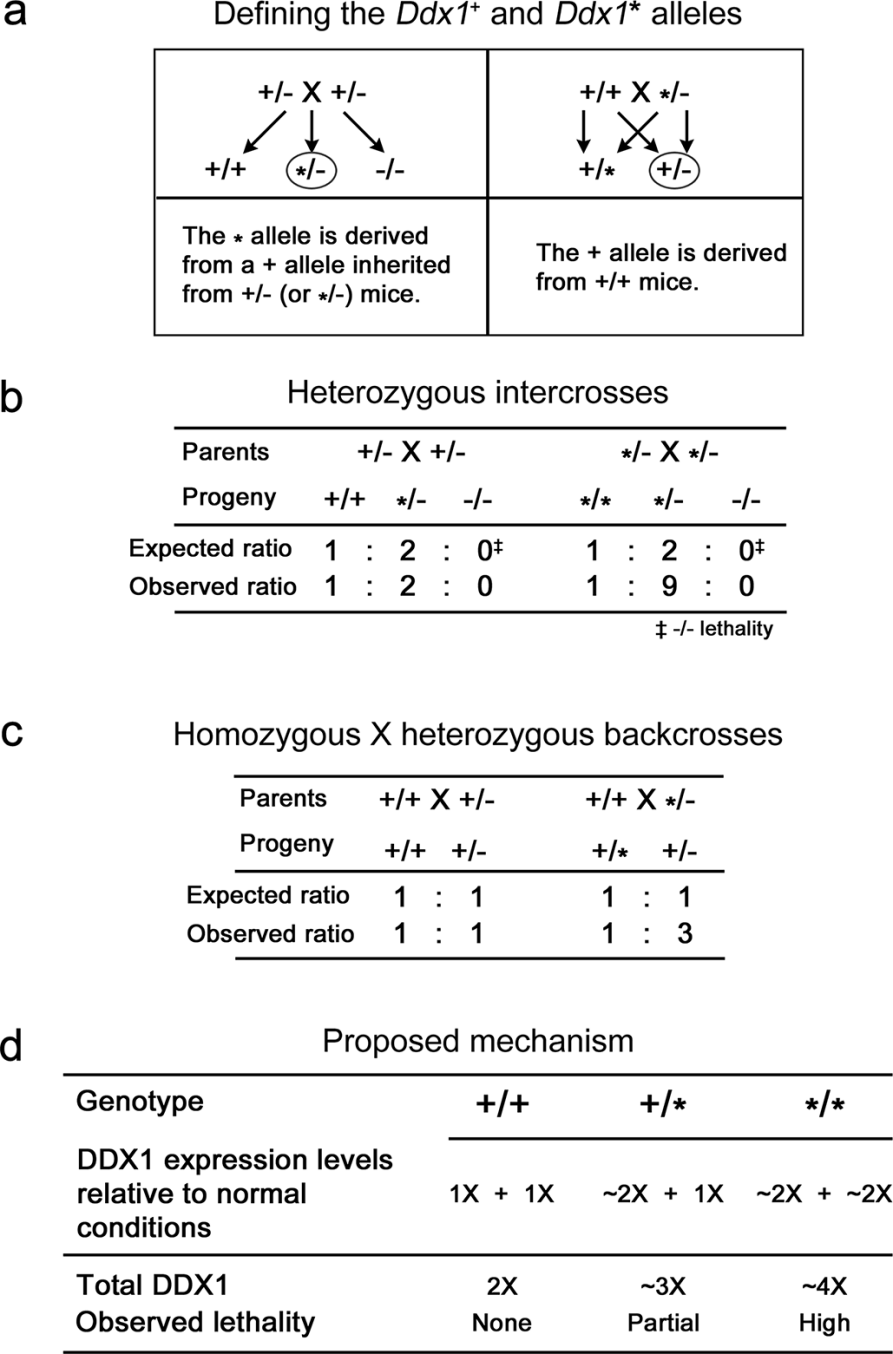
Inheritance model of the Ddx1* allele. (a) Depiction of the two types of wild-type alleles as determined by parental crosses. (b) *Ddx1*^+/−^ intercrosses produce the expected ratio of wild-type to heterozygote progeny, whereas Ddx1*^/− ^mice intercrosses produce an abnormal ratio of wild-type to heterozygote progeny. (c) *Ddx1*^+/−^ backcrosses produce the expected ratio of wild-type to heterozygote progeny, whereas partial wild-type lethality is observed in Ddx1*^/−^ backcrosses. (d) Proposed mechanism for wild-type lethality. Under normal conditions, each *Ddx1* allele produces 1X *Ddx1* RNA, resulting in a total of 2X DDX1 RNA and protein. Ddx1* alleles generate ~2X *Ddx1* RNA to compensate for inactivation of the mutant *Ddx1 allele*. Ddx1*^/+^ and Ddx1*^/^* mice are predicted to produce ~3X and 4X *Ddx1* RNA, respectively. This increase results in early embryonic lethality, with higher penetrance observed with increased levels of DDX1.

Unexpectedly, we observed significantly reduced numbers of wild-type mice when genotyping the progeny of *Ddx1* heterozygote crosses. Reduced numbers of wild-type mice were noted as early as the peri-implantation stage of development which occurs between E4.5 and E5.5 and remained constant at later stages of development suggesting stage-specific lethality. Through analysis of parental genotypes, we were able to identify two distinct populations of heterozygous mice: “abnormal” heterozygote mice (Ddx1*^/−^) which arose from heterozygote intercrosses (*Ddx1*^+/−^ X *Ddx1*^+/−^ or *Ddx1*^+/−^ X Ddx1*^/−^ or Ddx1*^/−^ X Ddx1*^/−^) and yielded reduced ratios of wild-type to heterozygote progeny, and “normal” heterozygous mice (*Ddx1*^+/−^) which arose from backcrosses (*Ddx1*^+/−^ X *Ddx1*^+/+^ or Ddx1*^/−^ X *Ddx1*^+/+^) and yielded the expected ratios of wild-type to heterozygote progeny (Figure 5a). Importantly, the wild-type lethality is not strain-specific as it was observed in both the FVB and C57BL/6 backgrounds. Thus, genetically identical heterozygous animals are able to distinctly and permanently modulate *Ddx1* expression at a very early developmental stage based on parental genotype. Although the mechanism of *Ddx1*^+^ to Ddx1* transition is unknown, it may be associated with the epigenetic reprogramming that takes place following MZT as the embryo proceeds to gastrulation^36^.

Two major modes of epigenetic inheritance have been described: paramutation inheritance and genomic imprinting. Paramutations occur when one allele modifies a second locus in a heritable manner. RNA mediated paramutations were first identified in plants, but have also been described in mice^37^,^38^,^39^. The first example of a paramutation in mice was at the *Kit* locus^40^. *Kit^+/−^* mice have a white-tail phenotype that is caused by loss of one copy of the Kit tyrosine kinase receptor gene. It was discovered that the white-tail phenotype could be maintained in wild-type (paramutant) *Kit^+/+^* offspring derived from *Kit^+/−^*heterozygote mice and all *Kit^+/−^* mice could generate paramutant *Kit^+/+^* offspring. Furthermore, the white-tail phenotype could be transmitted to the next generation when paramutant *Kit^+/+^* mice were mated with wild-type mice. Upon further investigation, it was discovered that miRNAs (miR-221 and -222) were being generated at high levels and inherited in subsequent generations through the oocyte or sperm, indicating *trans* rather than *cis* inheritance^40^. These abnormally high levels of miRNAs were responsible for modifying Kit levels from one generation to the next over the course of three generations, resulting in the white-tail phenotype. Two other paramutations were subsequently found to also be induced by miRNAs: *Cdk9* (miR-1) and *Sox9* (miR-124)^41^,^42^. While the phenotype associated with the Ddx1* allele shares some similarities with paramutations, the Ddx1* phenotype is limited to progeny which inherit the Ddx1* allele from *Ddx1^+/−^* intercrosses. Furthermore, in contrast to *Kit* paramutants which can be generated from *Kit^+/−^* backcrosses in addition to heterozygote intercrosses, mice with the Ddx1*^/−^ genotype are only observed in *Ddx1^+/−^* intercrosses and subsequent Ddx1*^/−^ intercrosses. Thus, our data indicate that, unlike RNA-mediated paramutations, the transgenerational phenotype associated with the Ddx1* allele is physically associated with the allele.

Genomic imprinting represents a non-conventional form of gene regulation and epigenetic inheritance that is *cis*-acting. Genomic imprinting is characterized by sex-specific changes to DNA methylation that occur during gametogenesis. Imprinted genes display mono-allelic expression, as one of the genes is silenced by methylation. As *Ddx1* expression is bi-allelic and the phenotype associated with the Ddx1* allele is sex-independent, genomic imprinting is not the mechanism regulating the modification of *Ddx1*. In an attempt to determine whether methylation marks might explain the Ddx1* phenotype independent of genomic imprinting, we sequenced bisulfite converted genomic DNA from wild type, *Ddx1^+/−^* and Ddx1*^/−^ mice. There were no changes in the methylation status of the single CpG island in the region surrounding *Ddx1*. Thus, we have yet to determine by what mechanism the Ddx1* phenotype is first generated and then maintained in order to be inherited by subsequent generations.

While we were able to clearly delineate the inheritance pattern underlying the lethality associated with the Ddx1*^/^* genotype, we can only speculate as to the underlying cause of lethality in Ddx1*^/^* embryos (Figures 5b-c). We propose that DDX1 protein levels are tightly regulated in the developing embryo, such that deviations from normal levels are lethal (Figure 5d). In support of this idea, attempts to generate lines of transgenic mice overexpressing DDX1 have been unsuccessful even in mice carrying multiple copies of the *Ddx1* gene (our unpublished data). Compensation in levels of DDX1 RNA and protein in heterozygous mice also indicates that DDX1 levels are tightly regulated. We propose that while heterozygous mice can easily compensate for reduced DDX1 RNA and protein levels by up-regulating DDX1 expression, downward compensation from Ddx1* alleles that are overexpressing *Ddx1* RNA does not occur. Thus, mice which inherit two compensating *Ddx1* (i.e. DDX1*) alleles die because of DDX1 overexpression (Figure 5d). It is still not clear if DDX1 over-expression is inherently lethal or causing aberrant development during early embryogenesis. The fact that some cancer cell lines can tolerate over-expression of DDX1^8^,^9^,^10^,^11^. is in keeping with disruption of developmental processes being the cause of lethality. Based on our data, modification of the wild-type allele in heterozygous mice is flexible for one generation, indicating that the “*cis*” mark is only added after fertilization in the second generation. As some lethality is observed in Ddx1*^/+^ offspring, we attribute this effect to a moderate increase in DDX1 levels that approaches the lethal threshold, such that embryos with acceptable variations in DDX1 levels survive, and embryos which surpass the threshold die.

In summary we found that DDX1 expression is essential for early mouse development, with *Ddx1*^−/−^ embryos failing to develop to the blastocyst stage. In the process of analyzing the progeny of heterozygote matings, we found that wild-type mice also die during development albeit at a later developmental stage than *Ddx1^−/−^* mice (pre E.6.5). In particular, our genotyping analyses indicate that the wild-type allele from *Ddx1^+/−^* intercrosses is physically marked through an unknown mechanism after the first generation of intercrosses. Our data indicate that DDX1 expression is tightly regulated during embryonic development, and that transcription of the wild-type *Ddx1* gene is up-regulated in *Ddx1*^+/−^ mice thereby compensating for loss of transcription from the mutant allele. We propose a model whereby inheritance of two wild-type *Ddx1* overexpressing alleles leads to embryonic lethality. While we have yet to establish the mechanism causing death during embryonic development, the transgenerational wild-type lethality phenomenon reported here does not appear to have been previously described in the literature and may represent a novel form of epigenetic inheritance.

## Methods

### Generation of *Ddx1* Mice

The mouse embryonic stem cell line (RRT447) containing an intronic gene trap within intron 14 of the *Ddx1* gene was purchased from BayGenomics. Chimeric *Ddx1* mice were generated by microinjecting RRT447 ES cells into C57BL/6 blastocysts. Male chimeric mice were mated to C57BL/6 females to obtain germ line transmission of the *Ddx1*^Gt(RRT447)RG ^allele (abbreviated as *Ddx1*). Two independent lines were obtained and characterized. To confirm *Ddx1* gene disruption at exon 14 and to ensure that there was a single insertion site of the *β-geo* reporter gene in our two lines, Southern blot analyses were carried out using ^32^P-labeled *β-geo* or *Ddx1* (exons 10-18) cDNAs. The *Ddx1* probe was generated by restriction endonuclease digestion of *Ddx1* cDNA with *EcoR*I and *Hind*III. The *β-geo* probe was generated with *β-geo* specific primers (5’: 5’-TTATCGATGAGCGTGGTGGTTATGC paired with 3’: 5’-GCGCGTACATCGGGCAAATAATATC).

To generate timed pregnancies, female mice were naturally mated to males. Females were examined for the presence of vaginal plugs over the course of 10 days. Mice with plugs were deemed to be at gestational stage E0.5. Plugged females were sacrificed at E3.5 and 6.5-10.5 to isolate embryos, which were subjected to genotyping by PCR as described below**.**

All experimental protocols related to animal work were approved by the Animal Care Committee, Cross Cancer Institute, Alberta Health Services (protocol BC11185). All methods were carried out in accordance with the approved guidelines of the Animal Care Committee.

### Genotyping of *Ddx1* mice

Genomic DNA was extracted from ear punches of weaned mice using the E.Z.N.A Tissue DNA Kit (Omega) according to the manufacturer’s instructions. Genomic DNA was collected from tails of P1 mice or from whole E6-10 embryos by digesting the tissue overnight in 100 µl Tris-EDTA-NaCl (TEN) buffer containing 40 µg/ml proteinase K (PK). The following day genomic, DNA was extracted using phenol/chloroform and precipitated with ethanol. E3.5 embryos were collected in 20 µl PCR buffer supplemented with 40 µg/ml PK. The embryos were digested at 55°C for 1 hour followed by 10 minutes at 90°C to inactivate PK.

Genotypes of E6 and older mice were determined by multiplex PCR in a 20 µl reaction volume containing 1 µl DNA template, 2 µl 10X PCR buffer (GE Healthcare), 0.4 µM of each primer (RGo60: 5’-CTGGGGTTCGTGTCCTACAA, RGo63: 5’-ATTAGGAACTGGGCATGTATC, and RGo65: 5’-AGCACTAGTAAGTACCTACAC), 250 µM dNTP mix and 0.2 µl Taq polymerase. The reaction was PCR-amplified under the following conditions: 94°C for 5 minutes followed by 35 cycles at 94°C for 1 minute, 60°C for 1 minute and 72°C for 1 minute followed by a final extension at 72°C for 10 minutes. The reaction mixture was separated on a 1.0% agarose gel in 1X Tris acetate-EDTA buffer.

Genotypes of blastocysts were analyzed by nested PCR. For the first round, we used 1 µl DNA template, 2 µl 10X PCR buffer, 0.8 µM of the following primers: RGo62: 5’-GATGGAGACAGTCCTGGTT paired with RGo66: 5’-CCAAGCTCCACTATTATCCC or RGo62 paired with RGo60, 250 µM dNTP mix and 0.2 µl Taq polymerase using the same amplification protocol described above. For the second round, we used 1 µl from the first round reaction, 2 µl 10X PCR buffer, 0.4 µM primers (RGo63/RGo65 for the RGo62/66 template or RGo63/60 for the RGo62/60 template), 250 µM dNTP mix and 0.2 µl Taq polymerase using the same amplification protocol described above.

### Statistical analysis

Expected groups were defined by applying the normal genotype ratio to the total number of progeny collected at each stage of development. Individual Fisher’s exact tests were performed between each expected group and the observed values to determine significant differences between the two groups.

### Western blot analysis

Protein was isolated from P1 brain tissue that had been previously flash frozen and stored at −80°C. Chilled lysis buffer (PBS containing 1% TX-100, 0.1% SDS, 1X Complete (Roche), 1 mM PMSF, and 1 mM DTT) was added to each sample. The samples were homogenized and centrifuged at 14,000 g for 10 minutes at 4°C before collecting the supernatant. Cell lysates (50 µg per lane) were electrophoresed in an 8% SDS-polyacrylamide gel. The proteins were transferred to PVDF membranes. Membranes were blocked with 10% milk in TBST (0.01% Tween-20) for 1 hour, then sequentially immunostained with anti-DDX1 (batch 2910; 1:5,000 dilution) and anti-actin (Sigma; 1:100,000 dilution) in 5% milk in TBST at 4°C overnight. The blots were subjected to anti-rabbit (for DDX1) and anti-mouse (for actin) secondary antibodies conjugated to HRP (Molecular Probes; 1:50,000 dilution) in 5% milk in TBST for 4 hours, followed by incubation with ECL reagent (GE) and exposure to X-ray film.

### Semi-quantitative RT-PCR

RNA was isolated from P0-3 mouse brains by homogenization in 1 ml Trizol (Life Technologies) as per the manufacturer's protocol. Complementary DNA (cDNA) was generated using Superscript II (Life Technologies) following the manufacturer's protocol using either oligo(dT)_12-18_ or random hexamer primers and 5 µg RNA. Semi-quantitative RT-PCR was performed in a 20 µl reaction containing 1 µl cDNA, 2 µl 10X PCR buffer (GE Healthcare), 0.4 µM of each primer pair (3’ *Ddx1*: sense, 5’-AGAATTATGTGCACCGGATC, antisense, 5’-GCACCAGAGGGTTAGAGT; *β-geo*: sense, 5’-CCTGTCCGGTGCCCTGAATG, antisense, 5’-GAAGAACTCGTCAAGAAGGCG; *β-geo-Ddx1* fusion:, sense, 5’-CTGAAGAGCTTGGCGGCGAAT, antisense, 5’-TTTGGATCCATGTACATCATCAGTTCTAAT; *Gapdh*: sense, 5’-ACGGCAAATTCAACGGCAC, antisense, 5’-GAGAGCAATGCCAGCCCC), 250 µM dNTP mix and 0.2 µl Taq polymerase. The reaction was amplified using the following conditions: an initial heating to 94°C for 5 minutes followed by 25 cycles (*Gapdh*) or 29 cycles (*Ddx1* or *β-geo*, or *β-geo-Ddx1 fusion*) of 94°C for 1 minute, 55°C for 30 seconds and 72°C for 1 minute followed by a final extension for 10 minutes at 72°C and a hold at 4°C. The reactions were electrophoresed in a 1% agarose gel to separate the amplified DNA.

### Quantitative real-time PCR

Total RNA was isolated from P0-3 brain and first-strand cDNA synthesized as above. The cDNA was amplified using TaqMan Fast Universal PCR Master Mix and gene-specific oligonucleotides (*Ddx1*, Mm01270541_m1; *Gapdh*, Mm99999915_g1) labeled at the 5′ end with the fluorescent reporter dye FAM (Life Technologies) (ABI 7900HT Fast Real-Time PCR System). The *Ddx1* oligonucleotide is 3’ to the LacZ insert. All cDNAs were run in triplicate, and the data were normalized using Gapdh.

### Bisulfite sequencing

1 µg genomic DNA prepared from *Ddx1*^+/+^, *Ddx1*^+/−^ and Ddx1*^/−^ mice was treated with sodium bisulfite using the EpiTect Bisulfite kit (Qiagen) using the manufacturer’s protocol with an additional cycle of denaturation for 5 minutes at 95^o^C followed by 2 hours at 60^o^C to ensure complete conversion. The converted DNA was amplified using 1 µl template, 10X PCR buffer (GE), 0.4 µM of each primer (sense, 5’-AAGTTTATAGGTTTTGAGTGAATTATT, antisense, 5’-CCAAACAAAACAACATCA TCTTTAC), 250 µM dNTP mix and 1 µl Taq polymerase in a 100 µl volume. The PCR reaction was electrophoresed in a 6% native acrylamide gel. The expected 700 bp band was cut out and electroeluted on dialysis tubing. The DNA was extracted with phenol and ethanol-precipitated. The purified DNA was ligated into the pGEM-T Easy (Promega) vector using the manufacturer’s protocol with overnight ligation at 16°C. *E. coli* DH5α competent cells were transformed with the ligated products and colonies selected by blue/white color selection. White colonies were selected for analysis and plasmid DNA purified using the QiaPrep Spin Mini plasmid kit (Qiagen)^43^. Plasmid DNA containing inserts were sequenced using the M13 reverse sequencing primer (5’-CAGGAAACAGCTATGAC). DNA sequences were then subjected to analysis by Bisulfite Sequencing DNA Methylation Analysis (BISMA) using default parameters and displayed using Methylation plotter^44^,^45^.

## Author Contributions

MH, EM and MB generated the data. MH and DG performed the data analysis and constructed the figures. MH, DG and RG wrote the main manuscript text. All authors reviewed the manuscript.

## Figures and Tables

**Table 1:** Genotypes of weaned progeny from heterozygous matings.

Strain	Total	Genotype by PCR		
		+/+	+/−	−/−
C57BL/6/*Ddx1*^+/−^	408	42 (10%)	366 (90%)	0
FVB/*Ddx1*^+/−^	292	34 (12%)	258 (88%)	0
